# The use of chatbots as interventions for mental health conditions: scoping review of systematic reviews and meta-analyses

**DOI:** 10.3389/fdgth.2026.1789599

**Published:** 2026-09-01

**Authors:** Khanh Dang Bao Tieu, Felix Agyapong-Opoku, Belinda Agyapong

**Affiliations:** 1 School of Medicine, University of Galway, Galway, Ireland; 2 Department of Psychiatry, University of Alberta, Edmonton, AB, Canada; 3 Department of Psychiatry, Dalhousie University, Halifax, NS, Canada

**Keywords:** Anxiety, Chatbots, conversational agents, depression, effectiveness, interventions, mental illness, meta-analysis

## Abstract

**Background:**

During the COVID-19 pandemic, the use of mental health chatbots received potential attention as a means of addressing the increase in demand for mental healthcare, which has become inaccessible due to a shortage of healthcare workers and lockdown. Even after the pandemic, chatbot versatility holds the potential to address the ongoing limited accessibility of mental healthcare due to a shortage of professionals, geographical challenges, cost, or stigma.

**Objective:**

This review examines the scope and nature of the systematic review and meta-analysis evidence on chatbot interventions for mental health.

**Method:**

This scoping review follows Arksey and O'Malley's five-step guide and was reported using the Preferred Reporting Items for Systematic Reviews and Meta-Analyses Extension for scoping reviews (PRISMA-ScR). The following databases were used to conduct all systematic literature searches: PubMed, Scopus, Medline, and Web of Science. Inclusion criteria include systematic reviews and meta-analyses, written in English, that focus on the use of chatbots as an intervention for mental conditions, with all primary articles being relevant. A total of 14 articles were relevant and included in the data extraction and analysis.

**Results:**

It was found that although current review-level evidence suggests potential short-term benefits, there was a discrepancy regarding the specific conditions under which chatbots are effective. Moreover, the use of chatbots for symptom improvement is still inconclusive, since there are issues with important aspects such as unclear risk of bias, lack of longitudinal studies, and lack of comparison with traditional therapy.

**Conclusion:**

There is potential for chatbot implementation as an intervention for mental conditions. However, this is still at its early stages of development and requires more research to improve communication quality and ensure safety, which is paramount when communicating with vulnerable patients. The findings of the review provide a useful foundation for informing clinical practice, policy, and future research.

## 1 Introduction

The definition of chatbots varies between different sources, with some limiting chatbots to text-based or voice-based systems (1), or AI-powered programs (2). However, one definition that stands out due to its high inclusivity is that chatbots are programs designed to mimic human conversation with human users (3). The early development of chatbots aimed to make them indistinguishable from a human in conversations (4). An example of this is ELIZA, a computer program capable of simulating a psychotherapist in conversations, serving as a research tool developed by Joseph Weizenbaum (5). In recent years, chatbots have become readily available on smart devices owned by billions of people, showing potential for use in mental healthcare (2). During the COVID-19 pandemic, various forms of chatbots were developed as intervention tools for mental illnesses, including conversational applications, avatar therapy, and companion bots (6). In this paper, we will explore chatbot interventions specifically targeting mental health symptom improvement, rather than chatbots’ use in general healthcare*.*

Chatbots can be differentiated based on their mechanism of function, such as rule-based, retrieval-based, and AI. Rule-based chatbots, the original form of chatbots, operate via a decision tree and predefined rules to generate responses (7). Retrieval-based chatbots evaluate the user's inputs as well as their context and determine the most appropriate response based on a set of predetermined responses, incorporating both rule-based and machine-learning elements (8). Finally, AI-based chatbots, which were recently developed but have become increasingly more common, are capable of interpreting users’ text and can self-generate responses based on that (9).

An estimation in 2019 stated that the global economic burden of mental disorders is about 5 trillion USD, and in high-income North America, this cost is estimated to be about 8% GDP, indicating mental health is a major issue (10). However, in many countries, significant barriers still limit access to mental healthcare (11). A recent scoping review in 2024 identified health system availability and complexity, working conditions, training/education, and patient accessibility as commonly reported themes of barriers to mental healthcare (12). This highlights the need to improve capacity and clinical training in the mental healthcare system. Moreover, in developing and more conservative communities, there is more stigma surrounding mental health, as it can be associated with shame, which prevents people from seeking support (13).

The implementation of chatbots in mental healthcare can address many of the existing barriers. For instance, the use of technology can make it easier for a hard-to-reach population, such as those in remote areas (7) or homeless individuals, who do not have access to mental healthcare but have comparable access to mobile technologies to their peers (14), to overcome the accessibility barrier. Similarly, patients with fears of stigma are less likely to engage in clinical mental healthcare but are more likely to seek support from a mental health chatbot (15). Moreover, chatbots are almost always available, as they can run 24 hours a day and communicate in different languages to better accommodate individuals (16). Furthermore, chatbots can improve mental health literacy, as they can provide comprehensible information and actionable guidance, which has been shown to encourage help-seeking behaviours (17) and lead to improved outcomes in self-care intention and behaviour (18). Regarding the therapeutic use of chatbots, one study (19) investigated the efficacy of the mental health chatbot “Woebot” at delivering Cognitive Behavioural Therapy (CBT) to college students who self-identified with symptoms of depression or anxiety. The results showed that the intervention group significantly reduced their depressive symptoms, measured by the Patient Health Questionnaire (PHQ-9), whereas the control group did not. Similarly, Nicol et al., 2022, investigated the therapeutic impact of chatbots on adolescents’ mental health (20). The primary analysis found that the intervention group experienced a decrease of 3.3 in mean PHQ-9, showing an improvement from moderate to mild symptom severity categories, and the control group showed no shift in symptom severity category. These studies highlight the therapeutic potential of mental health chatbots in improving symptoms of medical conditions by delivering active interventions, such as psychotherapy.

However, this technology is not without issues. For example, there are insufficient longitudinal studies investigating the impact of long-term exposure and interaction with mental health chatbots (2). Additionally, a 2024 study suggests that users should be cautious of forming an unhealthy virtual connection with chatbots, as such emotional interaction could worsen an individual's real-life communication and interaction (21). Many studies also highlight the risk of users’ data being sold to third parties, as there is limited privacy regulation regarding the use of chatbots, and medical confidentiality laws often do not cover chatbots (22). For instance, in the US, health data is created outside of the protection of the Health Insurance Portability and Accountability Act (HIPAA) (23). Many studies exist on the use of chatbots in mental healthcare and healthcare in general (24, 25) and are not confined to intervention, but also focus on other aspects of healthcare, such as diagnosis, resulting in high heterogeneity in the body of evidence. Thus, this scoping review aims to provide a comprehensive summary of the scope of evidence on chatbot interventions for mental health as well as identify any current research gaps.

## 2 Methodology

### 2.1 Study design

This scoping review of reviews was devised and performed according to Arksey and O'Malley's five-step guide for writing scoping reviews (26): Develop the research question, identify relevant studies, select articles, chart the data, summarises and report the results. This review was also performed in adherence to the Preferred Reporting Items for Systematic Reviews and Meta-Analyses guidelines for scoping reviews (PRISMA-ScR) (27). There is no previously published or registered protocol.

### 2.2 Develop the research questions

This scoping review of reviews’ objective is to determine the scope of the systematic review and meta-analyses evidence on chatbot interventions for mental health. The research question is “What is the scope and nature of the systematic review and meta-analysis evidence on chatbot interventions for mental health?”.

### 2.3 Identify relevant studies

This review used the following databases to conduct all systematic literature searches: PubMed, Scopus, Medline, and Web of Science. The search was completed on the 4^th^ of July 2025, and an updated search was completed on the 20^th^ of March 2026 using the same search terms. The search terms used were (Chatbot* OR Generative artificial intelligence OR Generative AI OR Conversational agent* OR Conversational artificial intelligence OR Conversational AI OR Natural language processing OR NLP OR large language model OR LLM OR Generative pre-trained transformer OR GPT OR Dialogue agent) AND (Mental health OR Psychological health OR Psychological wellbeing OR Mental wellbeing OR Emotional stability OR Mental wellness OR Mental illness OR Depression OR Anxiety OR Suicidal OR Eating disorder OR Post-Traumatic Stress Disorder OR Schizophrenia OR PTSD) AND (Systematic review OR Meta-analysis). The complete database-specific search strategy for PubMed has been provided in Supplementary material/Appendix 1.

### 2.4 Study selection

The relevant articles were selected in accordance with specific inclusion criteria as per our search terms, which are systematic reviews and meta-analyses, written in English, that focus on the use of chatbots as an intervention (symptom improvement) for mental illness, with each of the included reviews fully adhering to our inclusion criteria, focusing on chatbots as interventions for mental health, this was determined via the individual review own's inclusion criteria. For the purpose of this scoping review, chatbot intervention is considered to deliver active therapeutic content intended to produce measurable changes in mental health outcomes (symptom improvement, symptom reduction or improved coping), rather than solely providing general information. Changes in psychological symptoms over time were assessed using validated outcome measures, as recorded within each included systematic and/or meta-analysis, with reductions in symptom severity interpreted as symptom improvement. Positive effects of the intervention were evaluated by examining improvements in participants’ mental health, well-being, and resilience scores across assessment time points. In order to quantify this symptom reduction, primary articles used validated mental health scales. In contrast, information-providing chatbots are limited to disseminating static or non-personalised content without structured therapeutic components or mechanisms for behaviour change. Accordingly, inclusion criteria were restricted to chatbots incorporating evidence-based therapeutic techniques such as CBT-informed strategies with an explicit aim of intervention. Exclusion criteria include non-English studies, studies that do not exclusively focus on chatbots intervention for mental health such as mixed-scope reviews, and studies which are not a systematic review or meta-analyses.

**Figure 1:**
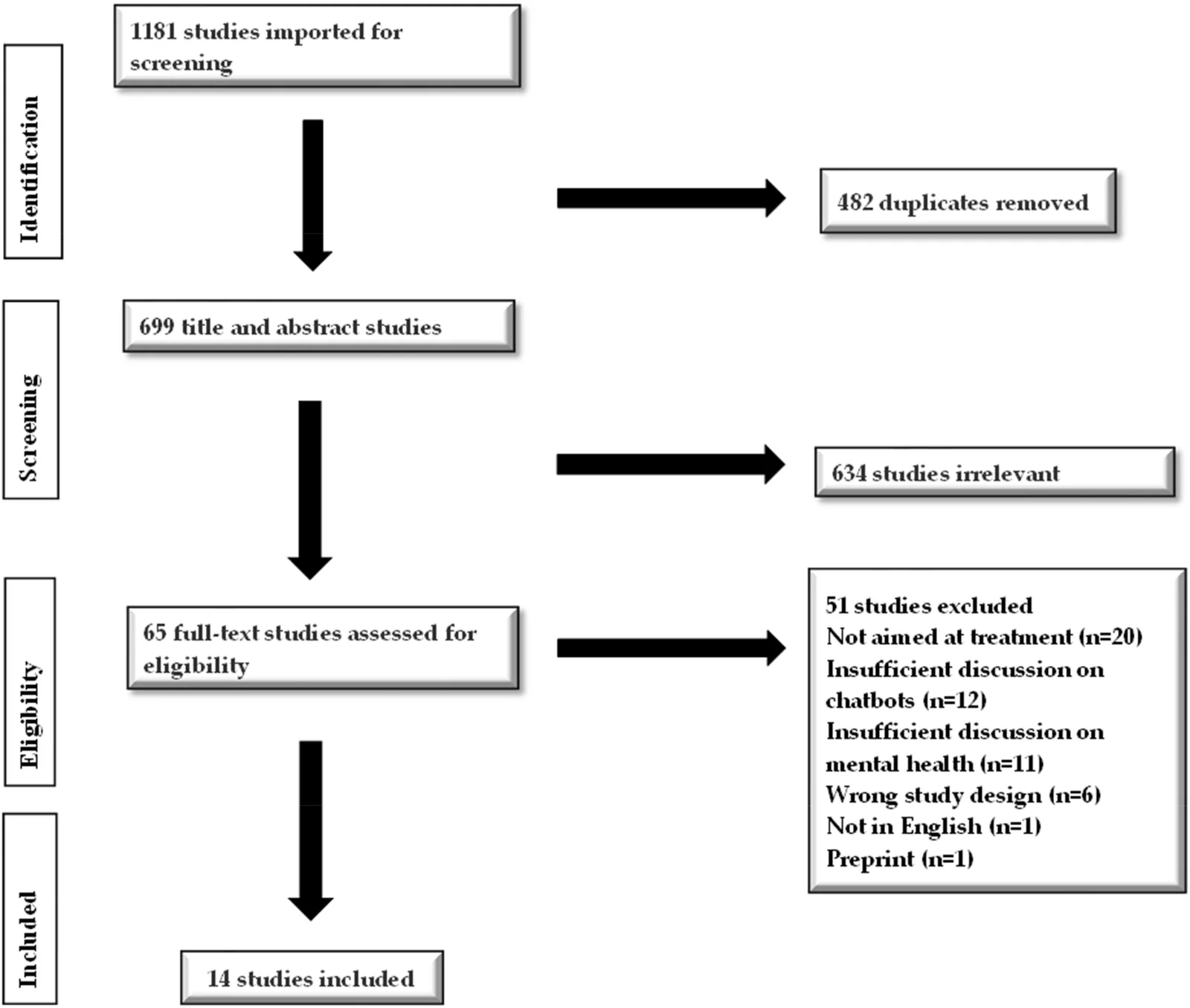
Prisma flow chart showing study selection process.

The studies were uploaded to Covidence (a web-based tool for systematic review) for title and abstract screening, followed by full text screening, conducted by 2 researchers independently. Following the updated search, 1181 studies were imported for screening, and 482 duplicates were removed, and 699 studies were screened for eligibility at the title and abstract stage. Of these, 65 studies were eligible for full-text screening, and 14 studies met the inclusion criteria and were included for data extraction and analysis. Conflicts during title and abstract screening were resolved by a third researcher, and conflicts during full text screening were resolved by the two researchers through discussion to reach a consensus. Visual representation of this process is presented in accordance with the PRISMA-ScR guidelines (27) as shown in Figure 1, PRISMA flowchart.

### 2.5 Data charting

Relevant data were organised into a table under the following categories: Author, publication year, Period, Number of studies included, Review Type, Countries of studies, Aim of the review, Participants characteristics, Risk of bias assessment, Types of chatbots, Duration, Delivery platform, Comparator conditions, Therapeutic approach, Outcomes/Findings, Recommendations. Risk of bias assessment were extracted from the included reviews.

### 2.6 Collating, summarising and reporting the results

This scoping review summarises the literature on the use of chatbots as an intervention for mental illness. All data were organised into tables and validated by 2 researchers and are presented in Table 1.

**Table 1:** Chatbots as intervention to improve mental health symptoms.

Author/Date	Period	Number of studies included	Review Type	Country of the primary article	Aim of the review	Participants characteristics	Risk of bias assessment	Type of chatbot	Duration	Delivery platform	Comparator condition	Therapeutic approach	Outcome/Findings	Recommendations
(30)	2016–2023	10	Meta-analysis	Romania, Spain and Scotland (*n* = 1) US (*n* = 5) China (*n* = 3) US, Malawi, and Brazil (*n* = 1)	Evaluates chatbots’ effectiveness in relieving symptoms of depression and anxiety in adolescents and young adults	Sample size: 18–1584 Age range: 15.7–35.3 The majority of participants are female	All studies achieve more than 7 positives in the quality checklist	-	4 weeks (*n* = 5) Not reported (*n* = 5)	-	-	CBT (*n* = 10)	Generally, chatbot interventions for depression are significantly more effective at reducing symptoms compared to controls. The effect size for depression was MD = −0.66 95% CI −1.09 to −0.23, *p* = 0.003 Regarding anxiety, overall, there were no significant differences in symptom reduction between chatbot interventions and control conditions. The effect size for anxiety was MD = −0.08 95% CI −0.56 to 0.40, *p* = 0.74	It is prudent to investigate developing different chatbots aimed at distinct mental disorders. Additionally, future research could diversify settings to increase the generalisability of the study.
(31)	January 2017–June 2024	10	Systematic review	-	Identifies the features of chatbots and their therapeutic effect; assesses their efficacy in the intervention of mental health disorders	Total sample size: 44773 Only 127 participants were in the control groups, as part of only 3 studies	6 studies achieved high, and 4 studies achieved moderate methodological quality in the quality checklist.	AI-driven (*n* = 10)	2 weeks (*n* = 1) 8 weeks (*n* = 1) 4 weeks (*n* = 1) Not reported (*n* = 7)	-	CBT e-book (*n* = 1) Waitlist (*n* = 1) Not reported (*n* = 8)	CBT (*n* = 10)	Overall, the three chatbots investigated —Woebot, Wysa, and Youper —are all capable of significantly improving participants’ mental health, including a reduction in depressive symptoms and substance use/craving.	Future research should focus on specific conditions that maximise chatbots’ effectiveness, such as symptom threshold, chatbots’ long-term benefits, and optimising users’ interactions.
(32)	Establishment of the database-August 6, 2024	14 (15 trials)	Systematic review and meta-analysis	-	Examine the effectiveness of chatbots for mental health in young people and the potential efficacy moderators.	Total sample size: 1974 Sample size range: 42–415 Age range: 12-25 1 trial focused on the clinical population 6 trials focused on the subclinical population 8 trials focused on the nonclinical population	1 study satisfied all 6 criteria 3 studies satisfied 4 criteria 5 studies satisfied 3 criteria 6 studies satisfied fewer than 3 criteria	Retrieval-based (*n* = 12) AI-driven: (*n* = 3)	12 minutes (*n* = 1) 20 minutes (*n* = 2) 1 week (*n* = 1) 2 weeks (*n* = 2) 2-4 weeks (*n* = 1) 4 weeks (*n* = 2) 6 weeks (*n* = 3) 8 weeks (*n* = 1) 12 weeks (*n* = 1) 16 weeks (*n* = 1)	Instant messenger platform (*n* = 6) Standalone app (*n* = 8) Web-based (*n* = 1)	Active control (*n* = 7) Information only (*n* = 4) Waitlist (*n* = 4)	CBT (*n* = 5) Method of levels (*n* = 2) Integrated approach (*n* = 5) Positive psychology (*n* = 1) Not reported (*n* = 1)	Chatbots demonstrated a medium to large effect on depression symptoms. Effect size is as follows: Hedges g = 0.61; 95% CI 0.35-0.86; *N* = 9; z = 4.61; *P* < 0.001 However, there was an insignificant effect for generalized anxiety symptoms (Hedges g = 0.06, 95% CI –0.21 to 0.32), stress (Hedges g = 0.002, 95% CI –0.19 to 0.20), positive affect (Hedges g = 0.01, 95% CI –0.24 to 0.27), negative affect (Hedges g = 0.07, 95% CI –0.13 to 0.27), and mental well-being (Hedges g = 0.04, 95% CI –0.21 to 0.29).	Chatbots might improve outcomes for anxiety by adopting more intensive behavioural strategies such as exposure therapy. Future research should investigate the long-term effects of chatbots on mental health outcomes.
(33)	2013–2018	13	Mixed-method systematic review	UK (*n* = 5) USA (*n* = 6) Sweden (*n* = 1) Japan (*n* = 1)	Review the use of chatbots as an intervention for mental health problems.	Total sample size: 1200 Sample size range: 14–454 Age range: 16–75 Participants were 70.3% female from studies that reported this data (12/13) Participants varied significantly in the severity of their mental problems	The studies’ quality ranged from 35% to 88%. The average quality score was 59%	-	1 session (*n* = 2) 1 week (*n* = 1) 2 weeks (*n* = 4) 2/4 weeks (*n* = 1) 4 weeks (*n* = 1) 4/8 weeks (*n* = 2) 1 month/30 days (*n* = 2)	-	Psychoeducation (*n* = 5) Active control with ELIZA (CA) (*n* = 2) Traditional therapy (*n* = 1) Correspondent to no treatment (*n* = 1) Waitlist (*n* = 1) Non-randomised no treatment (*n* = 1) No control group (*n* = 2)	CBT (*n* = 4) Method of levels (*n* = 2) Mindfulness-based stress reduction (*n* = 1) Structured communication enhancement strategy (*n* = 2) Eclectic interventions from multiple approaches (*n* = 4)	The findings were highly inconsistent. In some studies, chatbots have shown a varied range of improvements in mental health issues, from minor to significant, compared to control conditions. Whereas in other studies, the improvement seen in chatbots is not significantly different from that in the control conditions.	Future research should seek to improve chatbots’ flaws, such as repetitive and confusing responses. As well as comparing the modalities of chatbots and comparing chatbots with different modalities of intervention.
(1)	Establishment of the database –October 30, 2021, updated to May 1, 2022	32	Systematic review and meta-analysis	USA (*n* = 9) UK (*n* = 6) Sweden (*n* = 3) Japan (*n* = 2) South Korea (*n* = 2) Switzerland (*n* = 2) Canada (*n* = 2) Argentina (*n* = 1) China (*n* = 1) Ireland (*n* = 1) Netherlands (*n* = 1) Germany (*n* = 1) New Zealand (*n* = 1)	Summarise mental health chatbots’ traits, find evidence of efficacy, and analyse the statistically significant moderators of efficacy.	Total sample size: 6089 Sample sizes range: 19–2668 Average age: 36.32 Age range: 21.40–71.47 75.41% of the participants were female	8 studies had a low risk of bias 11 studies had some risk of bias 13 studies had a high risk of bias	-	≤1 day (*n* = 5) 1 hour–3/5 weeks (*n* = 1) 1 week (*n* = 2) 2-4 weeks (*n* = 11) 4-6 weeks (*n* = 2) 8 weeks (*n* = 4) 12 weeks (*n* = 1) 4/8/12/16 weeks (*n* = 1) 1 month (*n* = 1) 3 months (*n* = 2) 3 months–1 year (*n* = 1)	-	Active control (*n* = 9) Information control (*n* = 8) Attentional control (*n* = 1) Passive control (*n* = 13)	CBT (*n* = 17) Others, including patient-centred therapy, acceptance and commitment therapy, method of levels therapy, and problem-solving treatment (*n* = 15)	Chatbots were significantly better than control conditions in improving mental health in short-term in improving depressive symptoms (g = 0.29, 95% CI 0.20-0.38), generalized anxiety symptoms (g = 0.29, 95% CI 0.21–0.36), specific anxiety symptoms (g = 0.47, 95% CI 0.07–0.86), well-being (g = 0.27, 95% CI 0.16–0.39), general distress (g = 0.33, 95% CI 0.20–0.45), stress (g = 0.24, 95% CI 0.08-0.41), mental disorder symptoms (g = 0.36, 95% CI 0.17–0.54), psychosomatic disease symptoms (g = 0.62, 95% CI 0.14–1.11), and negative affect (g = 0.28, 95% CI 0.05–0.51. Long-term effects were statistically insignificant. Traditional text/voice chatbot had the largest effect size on generalised anxiety and life quality. Embodied chatbots (virtual humans) had the largest effect size for depression and stress. Virtual reality (VR) chatbots had the largest effect size for specific anxiety. Avatar showed the largest effect size for general distress.	Personalisation and empathic responses can significantly improve the efficacy of CAIs. Thus, this aspect of chatbots should be further developed in the future. The long-term efficacy of chatbots needs to be further explored. Future chatbot development should also aim to increase users’ active engagement in conversation, as engagement duration correlates with efficacy.
(34)	2007–2017	11	Systematic review and meta-analysis	Switzerland (*n* = 2) UK (*n* = 1) USA (*n* = 3) Germany (*n* = 5)	Examine the effectiveness of chatbots delivering psychotherapy to improve depressive symptoms in adults with depression or anxiety and evaluate the design features of chatbot-delivered psychotherapy.	Total sample size: 1099 Sample size range: 14–396 Participants are adults with a clinical diagnosis of depression or anxiety, and adults with subclinical disorders of depression or anxiety or both.	7 studies have an unclear risk of bias 4 studies have a high risk of bias	-	2 weeks (*n* = 1) 12 weeks (*n* = 3)	Online (*n* = 8) Offline (*n* = 3)	Treatment as usual (*n* = 4) Information only (*n* = 2) Waitlist (*n* = 5)	CBT (*n* = 2) Problem-solving therapy (*n* = 2) Others (*n* = 7)	Chatbot intervention results in significant improvement in depressive symptoms with a medium-sized effect [g = 0.54, 95% confidence interval (−0.66, −0.42), *p* < 0.001]. The overall size effect for anxiety was not provided. Embodied chatbots have a larger size effect.	-
(35)	January 2019–April 2024	12	Systematic review	-	Analyse the evidence of the effect of chatbot interventions on depressive symptoms.	Sample size range: 17–3629 Most studies have more female participants. Age range: 15.35–35.3 (only 5 studies reported age)	Risk of bias assessment was performed, with most studies having unclear risk of bias in key areas.	-	2 days (*n* = 1) 1 week (*n* = 2) 2 weeks (*n* = 2) 4 weeks (*n* = 3) 8 weeks (*n* = 2) 12 weeks (*n* = 1) 16 weeks (*n* = 1)	-	Uncontrolled (*n* = 3) Not reported (*n* = 9)	CBT (*n* = 4) CBT and talk therapy (*n* = 1) CBT, interpersonal therapy for adolescents, and elements of dialectical behavioural therapy (*n* = 1) Others (*n* = 6)	Most chatbots significantly improve depressive symptoms. However, some studies found no significant improvements or reported nonspecific improvements.	Future research should investigate the effect of chatbots on different populations. Additionally, methodological limitations and a more standardised methodological approach should be addressed and adopted, respectively, by future studies.
(36)	2014–2023	21	Systematic review and meta-analysis	Argentina (*n* = 1) China (*n* = 3) Italy (*n* = 2) Japan (*n* = 1) South Korea (*n* = 2) UK (*n* = 2) USA (*n* = 10)	Determine whether NLP models can improve depression and anxiety symptoms.	The majority of participants are adults and are female.	Regarding the randomised controlled trial (16). For studies on depressive symptoms, 9/16 have an overall low risk of bias, and 3 have an overall high risk of bias. For studies on anxiety symptoms, 8/16 have an overall low risk of bias, and 2/16 have an overall high risk of bias.	Rule-based (*n* = 10) AI-driven (*n* = 11)	-	-	Treatment as usual (*n* = 2) Conversational computer-based intervention (*n* = 5) Information, psychoeducation, or bibliotherapy (*n* = 8) Waiting list/no intervention (*n* = 8)	CBT (*n* = 16) CBT and dialectical behavioural therapy (*n* = 1) Others (*n* = 4)	AI-based NLP models were effective in reducing depressive symptoms (SMD 0.821, 95% CI 0.207-1.436; *P* < 0.001), but not effective in reducing anxiety symptoms (SMD 0.198, 95% CI –0.011-0.406; *P* = 0.1) Rule-based NLP models showed effectiveness in reducing both depressive (SMD 0.854, 95% CI 0.172-1.537; *P* = 0.01) and anxiety (SMD 0.347, 95% CI 0.116-0.578; *P* = 0.003) symptoms.	-
(37)	2016–2024	18	Systematic review and meta-analysis	Canada (*n* = 1) Switzerland (*n* = 1) Poland (*n* = 1) USA (*n* = 8) China (*n* = 3) Italy (*n* = 1) Japan (*n* = 1) South Korea (*n* = 2)	Evaluate the efficacy of chatbots in treating common mental health conditions such as depression and anxiety.	Total sample size: 3477 Sample size range: 18–1489 Age range: 18.78–40.29 (5 studies did not report)	Most studies seem to have a low to unclear risk of bias.	AI-driven (*n* = 18)	2 weeks (*n* = 2) 3 weeks (*n* = 1) 4 weeks (*n* = 7) 6 weeks (*n* = 1) 8 weeks (*n* = 4) 16 weeks (*n* = 1) 4 weeks, 8 weeks, 3 months (Follow-up) (*n* = 1) 8 weeks, 3 months (Follow-up) (*n* = 1)	Mobile apps (*n* = 9)	Treatment as usual (*n* = 2) Books (*n* = 4) Internet source (*n* = 1) Bibliotherapy (*n* = 1) VR therapy (*n* = 1) Information only (*n* = 1) Daily emotion rating (*n* = 1) No treatment (*n* = 2) Waitlist (*n* = 5)	-	Chatbots have a significant capacity to reduce symptoms of depression (g = −0.26, 95% CI = −0.34, −0.17; *p* < 0.001), similar to that of face-to-face therapy within a short intervention period, from 4 to 8 weeks. However, chatbots were less effective against anxiety (g = −0.19, 95% CI = −0.29, −0.09; *p* < 0.001), with a modest improvement in anxiety symptoms.	Further research could explore chatbots that employ a more diverse range of therapeutic theories (beyond CBT), such as mindfulness-based therapy and acceptance and commitment therapy. As well as focus on the long-term effectiveness of chatbots that promote and maintain good adherence.
(38)	2021–2025	15	Systematic review and meta-analysis	Japan (*n* = 1) China (*n* = 6) Turkey (*n* = 1) US (*n* = 4) Poland (*n* = 1) Argentina (*n* = 1) Italy (*n* = 1)	Evaluate the effectiveness of AI and rule-based chatbots in reducing depressive and anxiety symptoms	Age range: 14 – < 50 57% women, 43% men	Most studies have a low risk of bias, with some	Rule-based (*n* = 9) AI-driven (*n* = 6)	<4 weeks (*n* = 8) 4-8 weeks (*n* = 2) >8 weeks (*n* = 5)	-	Human (*n* = 4) - refers to psychological interventions provided by counselors, doctors, or nurses Book (*n* = 6) Blank (no intervention) (*n* = 5)	-	For depression, rule-based chatbots showed a small but significant improvement in symptoms (g = 0.266; 95% CI 0.020-0.512; *P* = 0.04), whereas AI chatbots did not achieve a significant effect (g = 0.407; 95% CI −0.734 to 1.550; *P* = 0.17). For anxiety, neither type of chatbots showed a significant effect in symptoms improvement, ruled based chatbots have an effect size of g = 0.147; 95% CI −0.073 to 0.367; *P* = 0.15 and AI chatbots have an effect size of g = 0.711; 95% CI −0.334 to 1.760; *P* = 0.13 The types of chatbots did not have a significant effect across different age groups (young, middle, and old)	Technological features that are significantly associated with efficacy should be identified and improved, thereby optimising future designs and precision. Future research should focus on methodological improvement to prevent uncertainty in research conclusions due to insufficient sample size.
(39)	2014–2023	10	Systematic review	USA (*n* = 3) Australia (*n* = 1) China (*n* = 2) Kenya (*n* = 1) UK (*n* = 1) Switzerland (*n* = 1) Ecuador, South America (*n* = 1)	Assess the impact of AI chatbots in improving depression and anxiety	Total sample size: 947 Sample size range: 41–213	71.67% of studies had a low risk of bias 15% of studies had unclear risk of bias 13.33% of studies had a high risk of bias.	AI-driven (*n* = 10)	2/4 weeks (*n* = 1) 1 week/1 month (*n* = 1) 16 weeks (*n* = 1) 4 weeks (*n* = 2)	-	Bibliotherapy control (*n* = 1) ELIZA program control (*n* = 1) Not reported (*n* = 8)	CBT (*n* = 6) Others (*n* = 4)	Chatbots achieved a noteworthy improvement in symptoms of depression and anxiety.	-
(40)	2020–2023	8	Systematic review and meta-analysis	Taiwan (*n* = 1) China (*n* = 3) India (*n* = 1) South Korea (*n* = 2) Japan (*n* = 1)	Examine the effectiveness of chatbots for improving mental health among people in Asia.	Total sample size: 921 Sample size range: 41–247 Age range: 18.8-44.5	12.5% of studies of low risk of bias 50% of studies were rated as having some concern 37.5% of studies have a high risk of bias	Rule-based (*n* = 2) AI-driven (*n* = 6)	Single session (*n* = 1) 1 week (*n* = 1) 3 weeks (*n* = 1) 4 weeks (*n* = 3) 16 weeks (*n* = 1) 6 months (*n* = 1)	Mobile app-based (*n* = 7) Online web-based (*n* = 1)	In-person mindfulness sessions (*n* = 1) E-book and chatbot (*n* = 1) Paper book (*n* = 3) No treatment (*n* = 2) Not reported (*n* = 1)	CBT (*n* = 5) CBT and emotional support (*n* = 1) Mindfulness (*n* = 1) Believed and goal-based (*n* = 1)	For depression, the effect of chatbots on symptom reduction was 0.46 (95% CI −0.76 to −0.16, *p* = 0.002). For anxiety, chatbots did not show any significant effect in symptom reduction with the effect size of −0.07 (95% −0.24 to 0.11, *p* = 0.45). For positive and negative affect, chatbots show no significant effects when compared with the control groups. Though there was a reduction in negative affect by 1.95 (95% CI −3.46 to −0.44, *p* = 0.01) when compared with no treatment. The certainty of evidence was reported as very low for depression, anxiety, stress, positive and negative affect. However, CBT chatbots showed significant between-group improvements in insomnia, attention deficit symptoms, panic disorder, social phobia and problem gambling in both when compared with control groups receiving bibliotherapy or no treatment. Mindfulness chatbots also reduce methamphetamine use in patients with methamphetamine use disorder.	Future studies should focus on methodological improvement that strictly adhere to reporting guidelines, including well-designed control groups, longer-term follow-up and adequate sample size, making it more suitable for clinical practice.
(41)	2017–2023	9	Rapid systematic review	-	Evaluates the effectiveness of chatbots in improving mental health outcomes and well-being among college students	Total sample size: 1082 Sample size range: 42–250	2 studies received a good PEDro score 2 studies received a fair score 5 studies received a poor score	AI-driven (*n* = 9)	1 session (12 minutes) (*n* = 1) 1 week (*n* = 1) 2 weeks (*n* = 1) 3 weeks (*n* = 1) 4 weeks (*n* = 1) 6 weeks (*n* = 1) 8 weeks (*n* = 1) 16 weeks (*n* = 1) 2-4 weeks (*n* = 1)	-	-	CBT-focused; mood tracking, goal setting, emojis (*n* = 1) CBT, mindfulness, psychoeducation (*n* = 1) CBT-based; depression assessment; empathetic responses (*n* = 1) Others (*n* = 6)	The majority of studies reported that chatbot intervention has statistically significant improvements in at least one mental health outcome. Among the primary articles, effect size ranges for depression from d = 0.09 to d = 0.83, for anxiety from d = 0.30 to d = 0.50, for stress from d = -0.72 to d = -0.33, and for well-being from d = -0.05 to d = 2.79.	Future chatbots must provide emergency responses to users with severe symptoms and referrals to human providers. Chatbots should be adjuncts, not replacements to traditional therapy, particularly during a crisis.
(42)	2020–2025	12	Meta-analysis	USA (*n* = 3) China (*n* = 2) UK (*n* = 1) Italy (*n* = 1) Finland (*n* = 1) Belgium (*n* = 1) Brazil (*n* = 1) South Korea (*n* = 1) Romania (*n* = 1)	Synthesising evidence focuses on the mental health chatbots’ function for children and adolescents.	Total sample size: 2961 Sample size range: 17–1715 Age range: 3–18 5 studies focused on the clinical population 6 studies focused on the non-clinical population 1 study focuses on both clinical and non-clinical populations	7 studies had a low risk of bias 5 studies were indicated as having some concerns	Rule-based (*n* = 8) AI-driven: (*n* = 2) Retrieval-based: (*n* = 2) (Woebot)	3 days (*n* = 2) 2 weeks (*n* = 1) 4 weeks (*n* = 1) 5 weeks (*n* = 1) 6 weeks (*n* = 1) 12 weeks (*n* = 2)	Web-based App (*n* = 2) Robot (*n* = 3) Smartphone (*n* = 5) Webpage (*n* = 1)	Telehealth CBT (*n* = 1) Distraction device (*n* = 1) Information (*n* = 4) Waitlist/no treatment (*n* = 6)	CBT (*n* = 7) CBT and rational-emotive behaviour therapy (*n* = 1) CBT and dialectical behaviour therapy (*n* = 1) CBT and interpersonal psychotherapy (*n* = 1) Acceptance and commitment therapy (*n* = 1) Health Action Process Approach (multi-theory model) (*n* = 1)	For negative mental health outcomes such as depression or anxiety, there is a significant, medium beneficial effect [ES = 0.468, SE = 0.184, t(10.8) = 2.54, *p* = 0.028]. For positive psychosocial well-being outcomes, there are no statistically significant effects [ES = 0.394, SE = 0.274, t(2.72) = 1.44, *p* = 0.255]. Audio chatbots showed significantly larger effect sizes compared to text-based chatbots, and significantly larger effects compared to multimedia chatbots.	A gap that should be addressed is that most trials had small samples, short durations, and relied mainly on self-report measures, with a lack of focus on treatment fidelity and user engagement. AI-driven chatbots can complement but not replace existing traditional mental health support.

Counts such as countries, participants characteristics, chatbot types, delivery platforms, comparator conditions, and therapeutic approaches are review-level occurrence counts and are subjected to overlapping.

The “-” symbol indicates that the data was not reported.

The sign of the effect size does not necessarily indicate the direction of symptom improvements, as this depends on certain factors such as calculation direction or scale orientation. Meaning both positive and negative effect sizes, could indicate symptoms improvements, provided that the confidence interval excludes 0.

### 2.7 Corrected covered area (Cca)

A corrected covered area was calculated according to the Pieper guide for assessing the degree of overlap between primary articles in the review of reviews (28). This involved creating a citation matrix of all unique primary studies (row) for the reviews (column) included. CCA can then be calculated using the formula $\frac{N-r}{(r\times c)-r}$, where N is the total number of primary articles presented, including overlapping, r is the number of unique primary articles, and c is the number of reviews (29). A CCA can be performed on all reviews as a whole; it could also be done between individual reviews.

## 3 Results

Overall, 14 articles were included in this scoping review of reviews. Table 1 gives details of the data collected from each systematic review or meta-analysis that utilised chatbots as an intervention for mental health, including therapeutic approach, outcome/findings, and recommendations.

### 3.1 Degree of overlap between primary articles

A total of 107 primary articles were included 195 times across 14 reviews, resulting in a CCA of 0.0633 or 6.33%, which is considered a moderate overlap (28), though on the lower end of this category (6–10). This value varies widely when comparing two individuals’ reviews, ranging from 0% to 44%. Again, this means that the data collected experienced some skewing, and due to the large range of CCA values, review pairs with high overlap, that is, those with a CCA value of equal or greater than 11%, according to Pieper et al. (28), will be considered when interpretation is made. Specific citations, matrix, and CCA calculation can be found in Supplementary material/Appendix 1.

### 3.2 Study characteristics

This scoping review of reviews comprises 3 systematic reviews (31, 35, 39), one mixed-method systematic review (33), one rapid systematic review (41), two meta-analyses (30, 42) and 7 systematic reviews and meta-analyses (1, 32, 34, 36–38, 40). The included studies were published between 2019 and 2026, with an increasing number of studies in recent years, starting from 2022 to 2025. Ten studies specify the number of countries from which the relevant articles originate (1, 30, 33, 34, 36–40, 42). Specifically, there are 55 occurrences of primary articles originating from North America, forty-seven from Europe, forty-three forty-two from Asia, six from South America, two from Africa, two from Oceania, and among these, there are 3 multinational primary articles (for multinational studies, each country is counted as a separate occurrence). When counting the unique countries, there are 12 from Europe, six from Asia (including Turkey), three from South America, two articles each from Africa, North America, and Oceania.

### 3.3 Targeted mental health conditions

The included studies covered a wide range of mental health issues; however, some studies focused on specific conditions. This includes depression, anxiety, stress, distress, substance use disorder, and insomnia. Table 1 discusses the conditions that individual studies focused on as part of the outcomes/findings section.

### 3.4 Chatbot technology

Nine studies reported the chatbot technology used in the primary articles (31, 32, 36–42). Among these, AI-driven chatbots (*n* = 75), retrieval-based chatbots (*n* = 14), and rule-based chatbots (*n* = 29) were reported. These studies (31) and (42) have a high degree of overlap with at least one of the others. Six of the highly overlapping studies reported either more AI-driven chatbots or only reported AI chatbots exclusively, with (32) being the exception and reporting mostly retrieval-based chatbots. This shows a divergent finding, suggesting an ambiguous definition between the different types of chatbot technologies. Similarly, it is important to note that (42) reported “Woebot” as a retrieval-based chatbot, whereas (31) consider “Woebot” to be AI-driven; this could be likely due to retrieval-based chatbots having elements of machine learning, and hence it is considered AI-driven, even though it is not generative. Four studies exclusively include AI chatbots. The specific AI technology was reported by only 4 studies. All 3 were generative AI in (32); all 6 were specified as a large language model (LLM) in (38); and all 2 were generative AI, with 1 LLM and 1 pre-trained LLM in (42) (36). reported two sentiment analyses, 2 natural language understanding, and 7 studies did not specify.

### 3.5 Comparator conditions

Twelve studies reported the comparator conditions in primary articles (1, 31–40, 42). This includes active control (*n* = 42), some of which was specified as traditional therapy (*n* = 13); information control such as bibliotherapy, books, or psychoeducation (*n* = 50); and passive control such as waitlist or no treatment (*n* = 57).

### 3.6 Intervention duration

The primary articles in the included systematic reviews and meta-analyses have a wide range of intervention durations, ranging from a 12-minute single session to up to 1 year. The reported intervention duration was highly heterogeneous and was categorised by this scoping review into the following categories: ≤ 1-week (*n* = 23), 2 to 4 weeks (*n* = 58), > 4 to 6 weeks (*n* = 10), > 6 to 8 weeks (*n* = 13), > 10 to 12 weeks (*n* = 8), and > 12 weeks (*n* = 10). Twenty-four primary articles were anomalous to this categorisation, these being 3/5-week (*n* = 1), 4/8-week (*n* = 2), < 4-week (*n* = 8), 4-8-week (*n* = 2), > 8-week (*n* = 5), 4 to 6-week (*n* = 2), 4/8/12/16 week (*n* = 1), 1 week/1 month (*n* = 1), 8-weeks/3-months (follow-up) (*n* = 1) and 4-weeks/8-week/3-months (follow-up) (*n* = 1). Table 1 outlines the conditions that individual studies focus on as part of the outcomes/findings section.

### 3.7 Therapeutic approach

Table 1 provides a detailed list of therapeutic approaches listed in the systematic review and meta-analysis included in this scoping review. Two studies did not report the therapeutic approaches in the relevant articles (37, 38). The most commonly used therapeutic approaches include at least some form of cognitive behavioural therapy (CBT), the method of levels, mindfulness, behavioural activation, problem-solving therapy, structured communication enhancement strategy, acceptance and commitment therapy, and an integrated approach. It was apparent that CBT is overwhelmingly the most common therapeutic approach used by chatbots, utilised in all 12 studies that reported therapeutic approaches and is the majority in 7 studies.

In Table 1, the therapeutic approaches “others” refer to therapeutic approaches that are not reported, non-specific or unconventional. For instance (34), reported the therapeutic approach for 7 primary articles as “mixed psychotherapy”, and (41) reported the therapeutic approach for 6 primary articles as, “Academic plus well-being integration; mindfulness, goal-setting”, “Integrative mental health support (psychoeducation, reminders)”, “Social robot; expressive movements; positive psychology”, “Social media-integrated (Facebook); symptom self-reflection”, “AI-driven; empathetic text/emojis; mood tracking”, and “Culturally adapted (India); academic stress management (diet, exercise, social)”, which this paper listed as “others”.

### 3.8 User characteristics

Six studies reported the gender ratio of participants, and in all of them, it was reported that most participants were female (1, 30, 33, 35, 36, 38). However, these studies have a high degree of overlap with at least one of the others. Nine studies reported the age of participants, which covers a wide age range, from 3 to 75 (1, 30, 32, 33, 35, 37, 38, 40, 42). Among these studies, there is less focus on the older age groups, as only 2 studies reported participants aged above 50, and most studies seem to focus on the younger population, from teenagers to middle-aged adults. However, excluding (42), the other 8 studies have a high degree of overlap with at least one of the others.

### 3.9 Key findings on interventions

Most studies found that chatbots have at least some positive effects on depression, anxiety, stress, distress, insomnia, or substance use disorder. However, it is important to note that there are inconsistencies between the findings of studies. For instance, three studies found that chatbots significantly improve depressive symptoms with effect sizes ranging from medium to small, but not anxiety (30, 32, 38), all of which have a high degree of overlap with at least one of the others. Whereas 3 other studies found that chatbots are effective for both anxiety and depression, with effects ranging from small to large for depression and small to medium for anxiety (36, 37, 41), with a high degree of overlap with at least one of the others. Interestingly, both (36) and (41) have a high degree of overlap with two of those that found chatbots to be effective only against depression and not anxiety, showing a divergence in findings regarding anxiety, despite their primary articles being similar. In one study, the effect sizes of chatbots on depression and anxiety were grouped as a medium-sized effect (42).Three studies (36, 37, 41) had a high degree of overlap with at least one of the others. Interestingly, both (36) and (41) have a high degree of overlap with two of those that found chatbots to be effective only against depression and not anxiety, showing a divergence in findings regarding anxiety, despite their primary articles being similar.

Moreover, one study (32) finds an insignificant effect of chatbots on stress and well-being. This contradicts the finding of (41), which shows chatbots have an effect size from small to large for both stress and well-being, whilst another study (1) showed that chatbots have a small effect on stress and distress. The article by Feng (32) has a high degree of overlap with (1) and (41), which shows a divergence in findings regarding stress and well-being.

Additionally (40), found that chatbots significantly improved symptoms of substance use disorder, attention deficit syndrome, social phobia, and gambling, even though the certainty of evidence is low for depression and anxiety, with statistically significant effects and statistically insignificant effects, respectively. In contrast (31) found that chatbots were both effective for depression and substance use disorder. These two studies do not have any degree of overlap (CCA = 0%).

There was no explicit inconsistency regarding the effectiveness of different subtypes of chatbots at symptom improvement, which was reported by three studies. Embodied chatbots were found to be more effective at improving depression in (1) and (34), which do not have a high degree of overlap. Traditional text/voice chatbots are reported to be more effective in the management of general anxiety, whereas virtual reality (VR) chatbots are more effective for specific anxiety, and avatar chatbots are more effective for improving symptoms of general distress (1). Audio chatbots were found to be more effective than text and multimedia chatbots at improving symptoms of depression and anxiety, but none improved well-being (42).

Two studies reported the effectiveness of different mechanisms (i.e., AI and rule-based) of chatbots at symptom improvement. It was reported that rule-based chatbots are more effective than AI chatbots, which showed no significant effect (38). Similarly (36), reported that rule-based chatbots were effective against both depression and anxiety, whereas AI chatbots were only effective against depression, but not anxiety. These two studies have a high degree of overlap. Another study (37) reported that chatbots’ as an intervention may be comparable to face-to-face therapy.

## 4 Discussion

This scoping review aimed to summarise the literature on the use of chatbots as an intervention for mental illness, as well as identify research gaps. It was found that although current review-level evidence suggests potential short-term benefits, there was a discrepancy regarding the specific conditions under which chatbots are effective. Moreover, the use of chatbots for symptom improvement remains inconclusive, as the overall risk of bias extracted from the included reviews was suboptimal, with many primary studies having a high or unclear risk of bias. Furthermore, there was considerable skewing, with similar findings from studies with a high degree of overlap, as well as divergent findings, where highly overlapping studies reached contradictory findings, suggesting that there might be methodological discrepancies among studies. Additionally, there are insufficient primary studies that compare chatbot intervention to face-to-face therapy, which highlights a gap in the current literature, as it means long-term clinical efficacy has not been demonstrated, which compromises the conclusiveness of chatbots’ effectiveness.

Based on the results, the vast majority of articles focused on depression and anxiety, though this number could be overrepresented due to the primary article overlap. It was clear that these two conditions still make up the majority. There are many other conditions for which the use of a chatbot as an intervention option has not yet been explored. Understandably, for many mental conditions, the use of psychotherapy might not be sufficient and often requires medication, so the use of chatbots, which aim to deliver psychotherapy, may not be adequate or suitable. However, chatbots may be extensively explored for conditions such as borderline personality disorder, insomnia, and others as adjunct psychotherapy. Unfortunately, there is little to no coverage by studies on these conditions, highlighting an important gap, as it shows the limited application of chatbots in mental health research. Future studies should address this by investigating the use of chatbots as an intervention in a more diverse range of mental conditions, other than depression and anxiety.

With recent advancements in AI technology, there is an increase in focus on AI-driven chatbots. This is partly suggested by the results, where a portion of reviews focus on AI chatbots, whereas no studies focus exclusively on the other types. However, this does not mean that there are more mental health AI chatbots than rule-based ones; though there are overwhelmingly more AI chatbots found in primary studies, this data is heavily skewed, since the majority of studies that report on chatbot technology have a high degree of overlap, and most reported an AI chatbot majority. Retrieval-based chatbots incorporate both rule-based and AI elements yet receive the least attention. However, this categorisation is ambiguous, as it could be interpreted as AI-driven. Future studies should aim to compare the therapeutic use of the different chatbot technologies, which are currently done by very few studies. In addition, there are very few mentions of emerging AI technologies such as generative AI or large language models (LLMs), which is unusual given their prevalence in recent years. Rather than the absence of generative AI implementation in chatbot technology, this seems to reflect an oversight of different AI technologies in chatbots, as this was reported in only 4 studies, whereas other studies seem to use AI as an umbrella term for such technologies. Thus, this highlights a gap in the literature where the diversity of AI technologies should be recognised and identified.

Regarding function, rule-based chatbots are noted to have a limited understanding of context and intention (9), making them less robust. AI-based chatbots are capable of analysing and interpreting a prompt to generate the most appropriate response. Retrieval-based chatbots are usually error-free with sufficient data and training, but have some weaknesses, such as stiff and non-human responses (8). The outcome from this review indicates that the efficacy of chatbots could be improved by personalisation and empathetic responses (1), which could be achieved through AI-based chatbots via machine learning. This enables more natural communication and should be used in future research (43). Despite this, AI-based chatbots are still capable of wrong interpretation. One crucial example is how many AI-based chatbots do not possess a sophisticated crisis detection mechanism (44). Thus, users might be misinformed or mistakenly redirected to crisis hotlines when they just need a conversation; this is critical as people suffering from mental conditions are highly susceptible, and any mistake could worsen the situation and might result in critical consequences (44). This warrants further improvement in AI chatbots, especially regarding safety regulations, as the lack of human control of AI technology could put users at risk. It was suggested that chatbots should not replace traditional therapy but rather should be used as an adjuvant to support it, particularly during a crisis (41, 42), especially considering that mental health chatbots are still at an early stage of development.

The overwhelming majority of chatbots utilised Cognitive Behavioural Therapy (CBT); again, this might be overrepresented due to the primary article overlap, but it was clear that the majority of chatbots utilise CBT as a therapeutic approach. However, CBT is a highly broad term and encompasses many forms of therapy, with potentially different outcomes. For example, it was suggested that intensive strategies such as exposure therapy, a form of CBT, could yield better outcomes for anxiety (32). Additionally, it was also recommended that future research should explore chatbots that employ a more diverse range of therapeutic theories, beyond CBT (37). This implies that different therapeutic approaches could influence the use of chatbots as an intervention, highlighting the potential for condition-specific therapeutic approaches for chatbots. Furthermore, the use of different types of chatbots could also yield condition-specific outcomes (1, 9, 34, 36). Thus, different chatbots may be developed for distinct mental disorders, as they often have different causes (30). Hence, the type of chatbot used as an intervention for a specific mental health condition could be deliberately chosen to address the specific cause of the mental condition to maximise intervention effectiveness.

There seems to be less focus on participants’ gender as this was reported by only 6 studies, and there is a high degree of overlap among them, suggesting a gap in the literature. Future research can investigate the use of chatbot intervention among males and females separately and compare the outcomes for each group. Similarly, even though participants’ age is reported in 9 studies, there is a high degree of overlap among 8 of them, which again shows a gap. Another study suggested that ease of use could influence intervention results (45), and technological literacy, which is generally higher among younger people, could affect user acceptability and feasibility. Invariably, it is important to investigate the use of chatbots among the respective groups, which was overlooked, as very few studies compared the use of chatbot interventions among different age groups. Elderly people often live alone or have limited mobility, which may reduce their access to mental health support, making the remote use of mental health chatbots appropriate. Therefore, there should be an incentive to encourage chatbot usage among this cohort, as well as to make it user-friendly for the elderly population.

Regarding countries of origin of primary articles, it was apparent that the majority of research on mental health chatbots was conducted in well-developed countries, and very few studies were conducted in developing countries. For instance, though North America has the highest number of primary article occurrences, studies were conducted in the US and Canada, with the overwhelming majority coming from the US. Likewise, in Asia, most primary articles originate from China, Japan, or South Korea, which are wealthier countries in the region. In contrast, only very few primary articles originate from Africa and South America, with 8 primary articles (not accounting for double counting) originating from 5 countries. With a limited number of primary articles originating from such regions, it is likely that the overlap between primary articles does not have any major implications. Therefore, future studies should prioritise studies conducted in South America and Africa to investigate the effects of mental health chatbots across diverse populations and to assess user acceptability. Although such research may be constrained by limited funding from less developed countries, it is particularly important given the reduced access to mental healthcare services and the persistent stigma surrounding mental health in these settings. Mental health chatbots may offer a valuable solution by providing low-cost, scalable and remotely accessible mental health support.

Concerning the publication year of the studies included in this review, there appears to be an increasing number of studies in recent years, starting from 2022. This can be attributed to the rise of chatbot technology during the COVID-19 pandemic, which aimed to improve access to mental healthcare (6) due to the rise in mental problems. One study reported a 27.6% increase in the prevalence of major depressive disorder and a 25.6% increase in the prevalence of anxiety disorder, which could be due to many factors, such as the rate of infection, the lack of mobility, lockdown, decreased social interaction, and closure of businesses (46). Chatbots enabled remote access through electronic devices, which many people own, allowing people to gain access to mental healthcare even during lockdown. Chatbots also provide a temporary substitution for a real therapist, which has assisted in facilitating an increase in demand for mental healthcare. For example, it can be a useful tool for stepped-care integration in mental health, as implemented in (47), where a mental health chatbot was used as an initial personalised intervention for mental health problems, before subsequent higher intensity manual intervention, during the pandemic. This saves healthcare professionals’ time and resources from having to intervene in the initial step, making it resource-efficient and scalable.

Consequently, the implementation of chatbots in clinical settings comes with numerous ethical and practical issues that need to be addressed by healthcare professionals. This includes informed consent regarding the collection and usage of patients’ data, with secure encryption and storage, and transparency regarding the chatbot's capability, non-human nature, and limitations (48). Furthermore, as previously discussed, there needs to be safety features for dealing with high-risk individuals, such as those with suicidal ideation, which could include an alert mechanism to detect high-risk phrases and refer patients to a hotline, a crisis response protocol where chatbots can guide users to professional help, or continuous monitoring of user data to seek patterns of deteriorating mental health and facilitate interventions (48).

Several research gaps remain regarding chatbots. For example, the long-term effects of using chatbots for mental health interventions are still unclear (49), with studies recommending further investigation (31, 32, 37). The findings of this study also support this, as the literature currently shows great heterogeneity when it comes to intervention duration, and most primary studies do not have sufficient intervention duration, as they may only last a few weeks, sometimes even minutes in extreme cases, compared to traditional therapy, which often takes months. Only 13 primary articles were reported to have a ≥ 3-month duration, which is especially concerning since overlapping articles means that this number could potentially be even lower. It is crucial that future studies match their duration with that of traditional therapy and also provide follow-ups. Many studies also do not account for the severity of mental illness, making it prudent to explore the potential outcome of chatbot intervention for different severities of a mental condition. However, this might be an ethical consideration, as subjecting people with severe mental illness to this kind of randomisation might have negative consequences. Moreover, active control groups only take up a portion of the comparator conditions in primary studies, and only 14 of those were classified as traditional therapy. This number could potentially be lower than the extracted data, due to overlapping in primary articles. This poses a problem since it is warranted that chatbot interventions be compared with the standard treatment, for their outcomes and role in mental healthcare to be accurately determined. Future research should aim to compare the outcomes, cost-effectiveness, user acceptability, and satisfaction of chatbots with in-person therapy (43). Additionally, the use of different subtypes of chatbots should be further investigated to reinforce the findings of (1, 34) and (36) that certain subtypes of chatbots are more effective for certain conditions. The adjunctive use of chatbots with in-person therapy is also understudied and can be investigated in future research (50). Some other concerns include confidentiality and privacy issues (45). Several studies have recommended that future research strengthen methodological quality, increase sample sizes and generalisability, extend study duration, and incorporate well-designed control groups (30, 38, 40, 42).

### 4.1 Limitation

The following limitations should be considered when interpreting the results of this study. Several systematic reviews and meta-analyses used overlapping articles, which may be caused by a relatively small body of evidence, making it inevitable for different reviews to use the same primary articles. Although the CCA calculation showed that there is only a small moderate level of overlap between primary articles, this still overrepresented certain data, such as countries of origin, participants’ characteristics, chatbot technology, duration, delivery platform, comparator conditions, therapeutic approach, and the mental health conditions that were targeted, which may affect interpretability and generalizability. Only full-text articles published in English that were classified as systematic reviews and/or meta-analyses were included in this study; consequently, studies outside these categories were excluded. The scoping review considered only systematic reviews and meta-analysis and hence there was no additional formal review-level quality appraisal. Additionally, there was heterogeneity in the interventions evaluated, and inadequate evidence on long-term outcomes, which may limit generalizability. Notwithstanding these limitations, this scoping review of reviews offers a vital perspective on the use of chatbots as an intervention for mental illness.

## 5 Conclusions

The scoping review offers important evidence that advances understanding of chatbots and their practical implications. There is potential for the implementation of chatbots as an intervention for mental conditions, but there is currently limited evidence to suggest the definitive effectiveness of chatbots on symptom improvement of mental conditions such as depression, anxiety, or substance use disorder, especially long-term. This is due to inconsistent findings among overlapping primary studies, which warrants a larger body of evidence to ensure reliability and offsets the existing inconsistency in findings; and limited long-term follow-up, which is a crucial step in demonstrating clinical viability. Additionally, there are insufficient direct comparisons with established treatments, thus showcasing that the literature on chatbot interventions for mental health is still at an early stage, and that chatbot technology still requires further development. However, the findings provide a useful foundation for informing clinical practice, policy, and future research. Continued investigation using longitudinal study designs and more robust methodologies will further strengthen the evidence and support the use of chatbots as adjunctive interventions to support mental health. This may warrant further research comparing chatbots with in-person therapy to better understand their role and potential application in mental healthcare. If future technological advancements demonstrate chatbots outcomes comparable to face-to-face therapy, mental health chatbots could represent a transformative development in mental healthcare delivery. Chatbots may be offered as an adjunct to traditional interventions, or potential options where access to the latter is compromised by financial constraints, geographic limitations, personal considerations such as stigma, or shortages in the mental health workforce.

## Data Availability

The original contributions presented in the study are included in the article/supplementary material, further inquiries can be directed to the corresponding author.
