# Supplementary material for: The use of chatbots as interventions for mental health conditions: scoping review of systematic reviews and meta-analyses
Source: Front Digit Health. 2026 Sep 1;8:1789599. doi: 10.3389/fdgth.2026.1789599 (PMC13574701; doi:10.3389/fdgth.2026.1789599)
Supplement: Supplementary file 1 [file Supplementaryfile1.pdf]

Appendix 1: PubMed Electronic Search Strategy

Search number	Query	Sort By	Filters	Search Details	Results
5	#1 AND #2 AND #3		from 1000/1/1 - 2026/3/20	(("chatbot*"[Title/Abstract] OR "Generative artificial intelligence"[Title/Abstract] OR "Generative AI"[Title/Abstract] OR "conversational agent*"[Title/Abstract] OR "Conversational artificial intelligence"[Title/Abstract] OR "Conversational AI"[Title/Abstract] OR "Natural language processing"[Title/Abstract] OR "NLP"[Title/Abstract] OR "large language model"[Title/Abstract] OR "LLM"[Title/Abstract] OR "Generative pre-trained transformer"[Title/Abstract] OR "GPT"[Title/Abstract] OR "Dialogue agent"[Title/Abstract]) AND ("Mental health"[Title/Abstract] OR "Psychological health"[Title/Abstract] OR "Psychological wellbeing"[Title/Abstract] OR "Mental wellbeing"[Title/Abstract] OR "Emotional stability"[Title/Abstract] OR "Mental wellness"[Title/Abstract] OR "Mental illness"[Title/Abstract] OR "Depression"[Title/Abstract] OR "Anxiety"[Title/Abstract] OR "Suicidal"[Title/Abstract] OR "Eating disorder"[Title/Abstract]	145

			OR "Post-Traumatic Stress Disorder"[Title/Abstract] OR "Schizophrenia"[Title/Abstract] OR "PTSD"[Title/Abstract]) AND ("Systematic review"[Title/Abstract] OR "Meta-analysis"[Title/Abstract])) AND (1000/1/1:2026/3/20[pdat])	
4	#1 AND #2 AND #3		("chatbot*"[Title/Abstract] OR "Generative artificial intelligence"[Title/Abstract] OR "Generative AI"[Title/Abstract] OR "conversational agent*"[Title/Abstract] OR "Conversational artificial intelligence"[Title/Abstract] OR "Conversational AI"[Title/Abstract] OR "Natural language processing"[Title/Abstract] OR "NLP"[Title/Abstract] OR "large language model"[Title/Abstract] OR "LLM"[Title/Abstract] OR "Generative pre-trained transformer"[Title/Abstract] OR "GPT"[Title/Abstract] OR "Dialogue agent"[Title/Abstract]) AND ("Mental health"[Title/Abstract] OR "Psychological health"[Title/Abstract] OR "Psychological wellbeing"[Title/Abstract] OR "Mental wellbeing"[Title/Abstract] OR "Emotional stability"[Title/Abstract] OR "Mental wellness"[Title/Abstract] OR "Mental	171

		illness"[Title/Abstract] OR "Depression"[Title/Abstract] OR "Anxiety"[Title/Abstract] OR "Suicidal"[Title/Abstract] OR "Eating disorder"[Title/Abstract] OR "Post-Traumatic Stress Disorder"[Title/Abstract] OR "Schizophrenia"[Title/Abstract] OR "PTSD"[Title/Abstract]) AND ("Systematic review"[Title/Abstract] OR "Meta-analysis"[Title/Abstract])	
3	"Systematic review"[Title/Abstract] OR Meta-analysis[Title/Abstract]	"Systematic review"[Title/Abstract] OR "Meta-analysis"[Title/Abstract]	562,643
2	"Mental health"[Title/Abstract] OR "Psychological health"[Title/Abstract] OR "Psychological wellbeing"[Title/Abstract] OR "Mental wellbeing"[Title/Abstract] OR "Emotional stability"[Title/Abstract] OR "Mental wellness"[Title/Abstract] OR "Mental illness"[Title/Abstract] OR Depression[Title/Abstract] OR Anxiety[Title/Abstract] OR Suicidal[Title/Abstract] OR "Eating disorder"[Title/Abstract] OR "Post-Traumatic Stress Disorder"[Title/Abstract] OR Schizophrenia[Title/Abstract] OR PTSD[Title/Abstract]	"Mental health"[Title/Abstract] OR "Psychological health"[Title/Abstract] OR "Psychological wellbeing"[Title/Abstract] OR "Mental wellbeing"[Title/Abstract] OR "Emotional stability"[Title/Abstract] OR "Mental wellness"[Title/Abstract] OR "Mental illness"[Title/Abstract] OR "Depression"[Title/Abstract] OR "Anxiety"[Title/Abstract] OR "Suicidal"[Title/Abstract] OR "Eating disorder"[Title/Abstract] OR "Post-Traumatic Stress Disorder"[Title/Abstract] OR "Schizophrenia"[Title/Abstract] OR "PTSD"[Title/Abstract]	1,141,486
1	Chatbot*[Title/Abstract] OR "Generative artificial intelligence"[Title/Abstract] OR	"chatbot*"[Title/Abstract] OR "Generative artificial	43,111

	"Generative AI"[Title/Abstract] OR "Conversational agent*"[Title/Abstract] OR "Conversational artificial intelligence"[Title/Abstract] OR "Conversational AI"[Title/Abstract] OR "Natural language processing"[Title/Abstract] OR NLP[Title/Abstract] OR "large language model"[Title/Abstract] OR LLM[Title/Abstract] OR "Generative pre-trained transformer"[Title/Abstract] OR GPT[Title/Abstract] OR "Dialogue agent"[Title/Abstract]			intelligence"[Title/Abstract] OR "Generative AI"[Title/Abstract] OR "conversational agent*"[Title/Abstract] OR "Conversational artificial intelligence"[Title/Abstract] OR "Conversational AI"[Title/Abstract] OR "Natural language processing"[Title/Abstract] OR "NLP"[Title/Abstract] OR "large language model"[Title/Abstract] OR "LLM"[Title/Abstract] OR "Generative pre-trained transformer"[Title/Abstract] OR "GPT"[Title/Abstract] OR "Dialogue agent"[Title/Abstract]	
